# Chroma+, a new automontage method of image background selection for Insects and other structurally complex objects

**DOI:** 10.3897/zookeys.795.26870

**Published:** 2018-11-05

**Authors:** Pavel Jakubec, Martin Novák, Jarin Qubaiová

**Affiliations:** 1 Faculty of Environmental Sciences, Czech University of Life Sciences Prague, Kamýcká 129, Praha – Suchdol, 165 00, Czech Republic Czech University of Life Sciences Prague Prague Czech Republic

**Keywords:** Automontage, entomology, methodology, photography, retouching, species digitalization

## Abstract

Obtaining taxonomic-grade images is a vital part of probably every present-day morphological study of insects, even though the task itself is perceived as a “necessary evil” due to high investment of both time and effort to produce representable images. Cleaning the background and making it appear as a solid color of known properties is probably one of the most time-demanding tasks. Several techniques have been developed to reduce the time requirement; the most convenient and cost-effective one presumably being the chroma isolation. This method uses a green background that can be isolated and conveniently replaced with another picture or solid color, as used in the film industry. However, the main drawback of this technique is spilling of color onto the object, which is unavoidable and can be corrected only by sacrificing the true color of the object to some extent. Our improved Chroma+ method is based on classical chroma isolation workflow and helps to overcome this problem by taking an additional image of the object with a neutral color background and applying a selection obtained from the chroma-isolated picture on it. This technique is, in terms of the resulting image quality, superior to classical chroma isolation, while the time difference between these two methods is negligible. Furthermore, it does not require any additional equipment (hardware or software), thus being accessible to both employed taxonomists, low budget laboratories, and enthusiasts.

## Introduction

A picture is worth a thousand words. This is very true in the field of comparative biology, a scientific discipline, which supports many others like community ecology, conservation biology, pest management, biosecurity, and biological control (Balke et al. 2013). All who work in these fields need to accurately identify species of interest and an image is the simplest and the most effective tool of how to pass the information, necessary for the task, on.

Techniques of photography have been evolving rapidly in the recent past and specifically macrophotography has seen an accelerated development in the last few decades. The rise of digital cameras opened many possibilities for creating perfect pictures and easily sharing them with others. These possibilities are still expanding beyond new horizons thanks to discoveries of new tools and work-flows. A perfect example of this phenomenon is the focus-stacking technique that enables widening of the available focus depth of the camera. This is achieved by merging several pictures with different focus distances together into one final sharp image, thus bypassing focus depth restrictions posed by the laws of optics (Thomson 2010). Due to these restrictions, it used to be impossible to create sharp images of very small objects (like insect specimens) in their entirety. However in the recent years, it has become a gold standard of good morphological descriptions to provide fully sharp image of the specimens that are discussed (Arino and Galicia 2005, Theodor and Furr 2009).

Once the perfect image is captured there is often an issue with the background. Many people prefer solid, well-defined colors (black, white, gray or another color according to their taste), which do not draw the attention of the viewer away from the photographed object and make placement of such images next to another easy; with backgrounds of the neighboring images blending together smoothly. This is, nevertheless, difficult to achieve directly with a camera. Overcoming these obstacles is often easier when using photo-retouching methods, where the image of the specimen is traced and the background is deleted or hidden by masking. That way, it can be replaced with an exactly defined RGB or CMYK color. Thanks to this technique, composing several images into one final figure is thus easy and straight forward.

Unfortunately, there is a price to pay. Common techniques used for background isolation are time-consuming and often largely rely on the user’s skill as one has to trace the whole contour of the specimen and erase the original background. This problem can be solved by using chroma isolation technique. The method uses a background that can be isolated (usually green or other color that is not occurring on the photographed object) and digitally replaced with another picture or solid color. Although chroma isolation can help in overcoming all of these issues, it often leaves colored halos around and on the object (Arino and Galicia 2005). These halos or color spills are practically impossible to remove from the image without affecting the original colors and therefore this method is not widely used in entomology.

However, chroma isolation, despite its obvious flaws, is a promising technique, being time efficient and fairly easy to use. In this paper we propose an improved chroma isolation method “Chroma+”, which deals with the main issue, the color spill, and further improves reliability of the specimen selection against its background.

## Methodology

The specimens used were obtained from the personal collection of the first author and from the collection provided by Jan Růžička (Prague). They were relaxed, unmounted, repositioned, and cleaned in an ultrasonic bath when needed, following standard protocols (Harrison 2012).

To obtain the pictures of our test specimens, we used a slightly modified Canon Cognisys setup (Canon EOS 550D with Canon MP-E 65 mm 1:2.8 1–5× Macro Photo Lens mounted on an automated macro rail for focus stacking (Cognisys StackShot)), which represents a standard setup in the field of macrophotography of insects (Brecko et al. 2014). For shooting image sequences of our specimens for consequent stacking, we used the Auto-distance mode, and the distance between steps (μm) was determined by magnification and F-stop used according to the depth-of-field table for Canon MP-E 65 mm 1:2.8 1–5× Macro Photo Lens, thus ensuring optimal number of images per stack.

**Figure 1.**
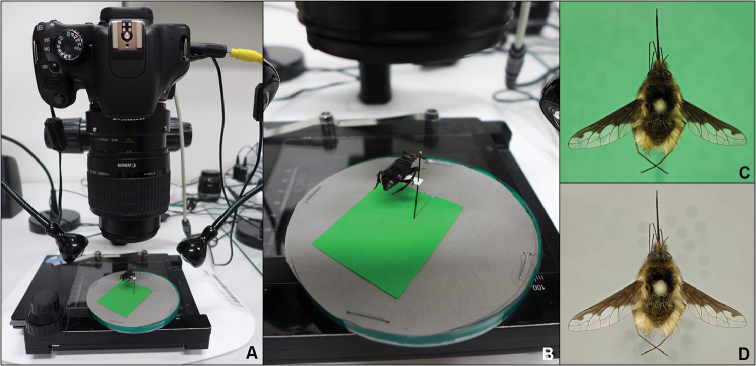
Chroma+ working space setup. **A** Overall setup of camera, light sources, and mounting plate for photographing the object **B** detail of mounting plate; green sheet can be removed by a simple pull (as it is cut near the pin) **C** resulting chroma-background image **D** resulting desired-background image.

The specimens were mounted on a pin and the setup allowed us to change the color of the background by simply placing colored paper in the space between the specimen and the polyurethane foam sheet (see Figure 1A–B). We made two image stacks for each specimen. The first one with a chroma background (Figure 1C) and the second one with a gray background (Figure 1D).

For focus-stacking the final images we used Zerene Stacker 1.04 software, as recommended by (Brecko et al. 2014) using PMax algorithm.

For isolation of the background we used GIMP 2.8.16 software (GIMP) and Photoshop CS5 (12.0) (PS) (versions for Windows). GIMP is a freeware and therefore a good choice for low budget laboratories and citizen scientists. It can also operate on wide variety of platforms like UNIX, MS Windows, Mac OS X. Photoshop is a purchased software, but provides more editing features with a larger level of sensitivity and sophistication of its tools.

We compared manual and semi-automated approaches using *Eraser* (PS) and *Background eraser* (PS) tools with automated methods *Color range* selection (PS) and *Refine edge* (PS) in classic chroma isolation and ultimately in our improved Chroma+ technique that can be deployed in both Photoshop and GIMP.

### Retouching workflow used in time efficiency testing

***a) Eraser***

1. Select Eraser tool and manually erase the background pixels or pixel groups. Accuracy is improved by making the tool smaller and by lowering the hardness (creating gradient between center of the tool and its edge).

2. Create new layer filled with desired color to serve as the new background.

***b) Background eraser***

1. Select Background Eraser tool (BE), set optimal value of the strength (lower values for backgrounds of similar color to the specimen, higher values for more differing backgrounds) and size of the tool.

2. Using this tool, erase the background around the circumference of the specimen. Several rounds of deleting with Background Eraser in different values may be needed. In cases where similar color of background and specimen require low values of BE, the background is often not deleted completely. Another round of BE with higher set value is thus required to get rid of the remnants of the background without simultaneously deleting parts of the specimen.

3. Delete the rest of the background with the simple Eraser tool, since it does not directly touch the specimen.

4. Create new layer filled with desired color to serve as a new background.

***c) Chroma isolation***

1. Load picture with chroma background (File -> Open...).

2. Define a chroma background using the Select Color Range (Select -> Color Range) in Photoshop or Select By Color tool (Select -> By Color) in GIMP. The selection is made by selecting specific shades of background and can be increased or subtracted in Photoshop by changing the level of Fuzziness within Select Color Range window as well as in GIMP by Shift + click or Ctrl + click. Selection is made instantly, so observe if the fit is improving or not and adjust accordingly.

3. (optional) Selection can be further improved by using other tools that allow edge feathering of the selection (Select -> Feather...) or shift it (Select -> Shrink... / Select -> Growth...).

4. The last step is using the created selection on the chroma image background. An option that allows good amount of flexibility is to use the selection for creation of the mask (in Photoshop, select the layer, and click New Layer Mask button in the Layers panel; in GIMP select the layer -> right click -> Add Layer Mask... -> Initialize Layer Mask to: Selection (check the box Invert mask) -> Add). Now the part of the image where the background should be is translucent and only the specimen with all its features should be visible.

***d1) Chroma+ (Photoshop)***

1. Load both pictures with chroma background and neutral background as layers of the same project and align them (Edit -> Auto-Align layers) using default settings.

2. Define a chroma background using steps 2 (optionally step 3) described in c) Chroma isolation method.

3. Once the chroma background is selected and the edge touching the specimen smoothed to a desirable level, remove it from the neutral background image by either directly deleting it or using a mask (step 4 in c) Chroma isolation method).

***d2) Chroma+ (GIMP)***

1. Load both pictures with chroma background and the neutral background (NB) (File -> Open as Layers...) and align them with Image Registration plug-in (Tools -> Image Registration...).

2. Define a chroma background using steps 2 (optionally step 3) described in c) Chroma isolation method.

3. Once the chroma background is selected and the edge touching the specimen smoothed to a desirable level, remove it from the neutral background image by either directly deleting it or using a mask (step 4 in c) Chroma isolation method).

## Results

### Manual and semi-automated selection

The manual and semi-automated approach for creating a selection of the object of interest is a straight forward method. The retoucher has to trace the whole contour of the object and delete everything that is not a part of it. Usually, the *Pen* tool or *Eraser* tool are used, but Photoshop also offers more sophisticated tools like the *Background eraser*, where the user selects the threshold and the tool itself determines what the object is and what the background is. This is what we consider a semi-automated approach.

**Figure 2.**
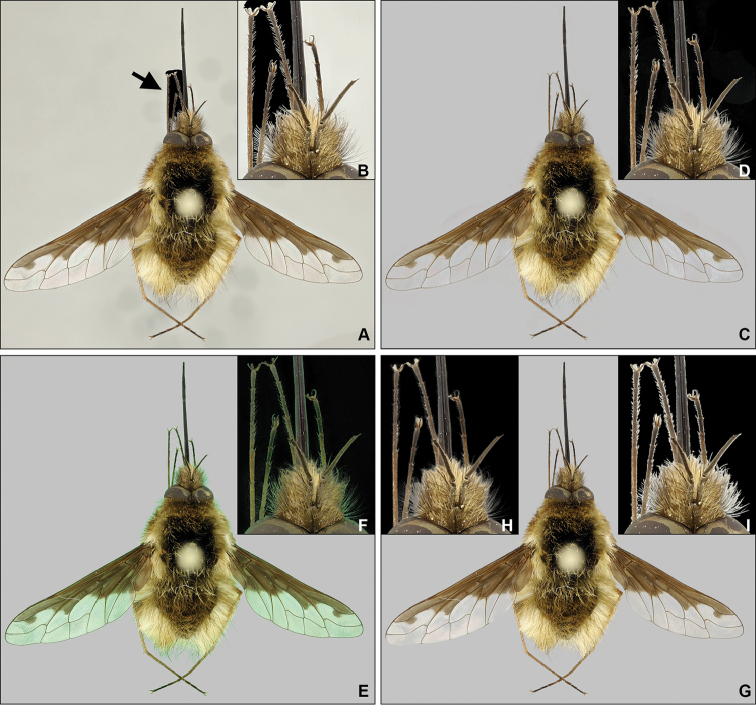
Comparison among methods of background selection. **A** Manual Eraser method; resulting image, arrow showing the amount of background removed after 45 min. **B** Manual Eraser method; magnified detail of removed background. **C** Background Eraser method; resulting image. **D** Background Eraser method; magnified detail showing imperfections in cropping and artefacts in the background. **E** Chroma method; resulting image, object with green halo. **F** Chroma method; magnified detail. **G** Chroma+ method, resulting image (Photoshop). **H** Chroma+ method; magnified detail after using Photoshop. **I** Chroma+ method; magnified detail after using GIMP.

The *Eraser* tool was used to create Figure 2A–B and the image is a result of 45 minutes of work. In comparison, the semi-automated approach using the *Background eraser* tool resulted in a completely uniform background around the whole object (Figure 2C–D) in the same amount of time.

From a qualitative perspective, it is worth mentioning that the *Background eraser* tool is unable to perform as a stand-alone tool and some parts of the background had to be removed by the basic *Eraser* tool as the semi-automated tool is unable to distinguish the background from the object when they are of similar color or brightness.

We did not use the *Pen* tool despite the fact that it is a much praised tool by professional retouchers. Due to the complex shape of our object, the time spent would be similar to the manual *Eraser* approach, meaning tedious work with doubtful results.

### Automated selection

**Chroma isolation.** The time required for making a selection and refining it consists of only several minutes. However, a background that is not fully out of focus is unsuitable for this method as it is necessary to deal with scratches and dust spots on its surface. If the specimen is pinned, being at a sufficient distance from its background, the selection can be done in less than a minute.

**Figure 3.**
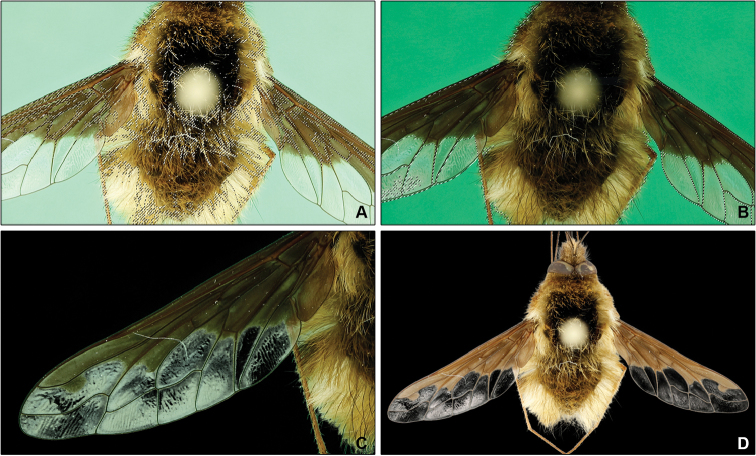
Limitations and troubleshooting. **A** Excessive color selection caused by a low-contrast chroma image **B** correct color selection due to a high-contrast chroma image **C** disadvantage of simple chroma method; green halo over object and color spill in translucent areas **D** unnatural halo around the object, caused by significant difference between photographed and assigned backgrounds.

The quality of the results is strongly limited by the properties of the specimen itself. If the object is covered with fine hairs along the edges or has translucent parts like wings, the color of the background will spill over or under these structures (Figures 2E–F and 3C) and removing it without affecting the original coloration of the specimen will become impossible. Even objects with round edges are prone to color spill as can be seen on Figure 2E.

We found two main advantages of this method, the first one being indisputably its speed, while the second is the surprising simplicity of the whole selection process. However, the above mentioned quality issues make this method practically useless for production of high quality images for publications.

**Chroma+.** Both speed and efficiency of the chroma isolation method are clearly superior in comparison to all other methods, but the quality issues are limiting it in its usefulness for descriptive morphology. The Chroma+ technique is based on the fact that we are working with stationary objects and the background can be changed between the shots. Therefore, we can create two pictures; one with a chroma background (green, blue, red, etc.) and the second with a background similar to the one that we would like to have in our final image (usually white, black, or gray). The object in these two images should be perfectly aligned (this can be fixed to some extent by using software automated alignment tools), thus when opened in the graphical software as layers, the chroma background image can be used to create the selection in the same way as in chroma isolation method. This selection is then used on the second image with the desired background color. In that way we can obtain the selection around the object without any color alternation caused by the spill from the chroma background.

From a qualitative perspective this approach enables dealing with complex edges and is comparable to the manual and semi-automated methods (Figure 2G–I). Time-wise, this approach is slower than chroma isolation, because you have to make a second image. However, when we compare the Chroma+ with methods that are producing images of similar quality, it easily surpasses them without much difficulty. The various differences between these methods can be observed in Table 1.

**Table 1.** Comparison of various approaches for isolating an object from its background performed by the same person on the same specimen of Bee fly (Bombyliidae) (N = 1). See Figure 2 for results.

Traits	Manual	Semi-automated	Chroma isolation	Chroma+ (PS)	Chroma+ (GIMP)
**Photo acquisition time (in minutes)**	10	10	10	15	15
**Creating the selection (in minutes)**	45+ *	45	1	1	5
**Total time**	55+	55	11	16	20

* see Figure 2A–B for actual extent of selection accomplished within 45 mins.

## Discussion

Chroma isolation represents one of the automated selection methods where the human element is supervising the final result, assisting the computer program only by choosing the appropriate shades of the background that should be selected. Both Photoshop and GIMP offer tools (e.g., Color Range tool (PS) and Select by Color tool (GIMP)) that allow isolation of the background based on a specified color range.

The Chroma+ technique inherited one crucial trait of its predecessor and that is its simplicity. Here, the user’s input in creating the selection is minimal, thus facilitating more consistent results among the users. This distinguishes it from manual and semi-automated methods where the quality of the results is highly dependent on the user’s experiences and skills.

Chroma isolation is often called “green screen”, but green is not the only color that can be used. We highly recommend using colors that are not present on any part of the object. Therefore, a green object can be photographed against blue or red backgrounds.

### Possible limitations and troubleshooting of Chroma+

One of the possible issues with the two-picture approach of Chroma+ is the position shift that occurs due to the physical interaction with the specimen or camera. The shift causes a misalignment between the two pictures and the mask created over the chroma image will not fit on the second image with the desired background. This issue can be greatly mitigated by avoiding physical contact with both the specimen and the camera. When this is not possible, alignment of the two images using graphical software is an option. However, such a solution is suitable only for a horizontal shift in position and the tool is incapable of correcting the angular shift.

Isolation of the object is rarely perfect and we are striving to a selection that is as close as possible to the object itself without cropping it. Ultimately, there is always a small halo (often in range of a few pixels) around the object. When the object is photographed on a white background this halo can be observed when we replace the background of the image with a dark color such as black or dark gray (Figure 3D). Although, it may be seen as a flaw of the selection function, it is an inevitable result of the background color spill over the object. Cropping it would be a mistake resulting in alternation of the specimen shape and possible loss of some features such as fine hairs. To prevent this issue, we suggest using at least a similar color of the background as the one you desire in the final image. This will allow you to blend in the halo and the background in an inconspicuous way.

The color selection of the chroma background appears to be more accurate on slightly underexposed images as the contrast between the object and the background increases. This is often used in the classical chroma isolation technique, but the limited dynamic range of the camera sensor dictates how much underexposure can be achieved in one image without compromising quality of the result (underexposed image of the specimen is unacceptable). The Chroma+ allows you to underexpose the first image as much as necessary, since it is only used in creating the selection and will not show up in the final image. This allows full freedom to create enough contrast between the object and the chroma background (Figure 3A–B). We achieved the best results with contrast levels making the object appear almost like a silhouette.

The selection can be adjusted with other tools available in the used software. You can add or subtract some areas where the selection algorithm did not work properly. There are often translucent parts (e.g. wings, elytra, etc.) where the color selection tool has difficulty differentiating them from the shades of the chroma background. A wide range of tools is available in each program such as the *Quick selection* tool (PS), *Free selection* tool (GIMP) or *Background eraser* tool (PS). After the selection is made, you are additionally allowed to adjust the hardness of the transition between the object and background. We prefer a slightly softer look of the edges as they seem to blend with the background more naturally. This look can be achieved by using the *Refine edge* tool (PS) or applying the feather function (GIMP) on your final selection (feathering 3 pixels is usually more than sufficient).

In conclusion, the Chroma+ method can be performed with basic macrophotography equipment, using freeware such as GIMP. This makes our method a viable option for both employed taxonomists in low budget laboratories and insect photography enthusiasts.
